# Small Nerve Fiber Pathology in Critical Illness

**DOI:** 10.1371/journal.pone.0075696

**Published:** 2013-09-30

**Authors:** Nicola Latronico, Massimiliano Filosto, Nazzareno Fagoni, Laura Gheza, Bruno Guarneri, Alice Todeschini, Raffaella Lombardi, Alessandro Padovani, Giuseppe Lauria

**Affiliations:** 1 Department of Anesthesia and Critical Care Medicine, Section of Neuroanesthesia and Neurocritical Care, University of Brescia at Spedali Civili, Brescia, Italy; 2 Department of Clinical Neurology, Section for Neuromuscular Diseases and Neuropathies, University of Brescia at Spedali Civili, Brescia, Italy; 3 Department of Clinical Neurophysiology, University of Brescia at Spedali Civili, Brescia, Italy; 4 Neuromuscular Diseases Unit, “Carlo Besta” Neurological Institute, IRCCS Foundation, Milan, Italy; Dalhousie University, Canada

## Abstract

**Background:**

Degeneration of intraepidermal nerve fibers (IENF) is a hallmark of small fiber neuropathy of different etiology, whose clinical picture is dominated by neuropathic pain. It is unknown if critical illness can affect IENF.

**Methods:**

We enrolled 14 adult neurocritical care patients with prolonged intensive care unit (ICU) stay and artificial ventilation (≥ 3 days), and no previous history or risk factors for neuromuscular disease. All patients underwent neurological examination including evaluation of consciousness, sensory functions, muscle strength, nerve conduction study and needle electromyography, autonomic dysfunction using the finger wrinkling test, and skin biopsy for quantification of IENF and sweat gland innervation density during ICU stay and at follow-up visit. Development of infection, sepsis and multiple organ failure was recorded throughout the ICU stay.

**Results:**

Of the 14 patients recruited, 13 (93%) had infections, sepsis or multiple organ failure. All had severe and non-length dependent loss of IENF. Sweat gland innervation was reduced in all except one patient. Of the 7 patients available for follow-up visit, three complained of diffuse sensory loss and burning pain, and another three showed clinical dysautonomia.

**Conclusions:**

Small fiber pathology can develop in the acute phase of critical illness and may explain chronic sensory impairment and pain in neurocritical care survivors. Its impact on long term disability warrants further studies involving also non-neurologic critical care patients.

## Introduction

Survivors of critical illness may suffer from prolonged disability [1]. Several causes contribute to this so-called “post-intensive care syndrome”, which is defined as new or worsening impairment in physical, cognitive, or mental health status arising after critical illness and persisting beyond acute care hospitalization [2]. Critical illness polyneuropathy affects sensory and motor fibers innervating limb and respiratory muscles [3] and is a prominent cause of persisting physical disability [4], leading to difficulty in weaning the patient from mechanical ventilation and flaccid tetraparesis [5]. Sensory nerve impairment is usually reported after intensive care unit (ICU) discharge [5,6]. Stocking and glove sensory loss, feeling of cool extremities, numbness, tingling, and neuropathic pain symptoms including hyperalgesia and burning have been described at long-term follow-up of ICU survivors [5,6]. In some patients, pain can be extremely intense, precluding effective rehabilitation [7]. In a recent series of 16 patients with acute respiratory distress syndrome evaluated at 6 to 24 months after ICU discharge, 6 patients reported variable combinations of pain, numbness, tingling, diffuse weakness, cool extremities, and impaired exercise ability [6] in the absence of abnormalities at nerve conduction study (NCS). The lack of correlation between clinical and electrophysiological findings suggested a small fiber neuropathy [6]; however, skin biopsy, which is required to document the degeneration of intraepidermal nerve fibers (IENF) and to define the diagnosis [8], was not performed and therefore the hypothesis could not be confirmed.

Degeneration of IENF has been described in small fiber neuropathy (SFN) of different etiology [9,10] and in other neuropathies dominated by neuropathic pain, including those with predominantly large fiber involvement such as Guillain-Barré syndrome [11-14] and chronic demyelinating polyradiculoneuropathy [15]. Most recently, small fiber dysfunction has been also described in fibromyalgia [16]. The diagnosis of SFN is confirmed by the quantification of IENF density at the distal site of the leg, based on age and gender-adjusted normative reference values [17]. When a more diffuse and non-length depended process is suspected, such as in sensory neuronopathies [18], or in acute or chronic demyelinating neuropathies [15,19], the IENF density should be examined also at a proximal site of the body, such as the thigh or the trunk.

We hypothesized that critically ill patients may suffer from small nerve fiber pathology as part of the generalized inflammatory process leading to multiple organ dysfunctions and failures. To address this hypothesis, we prospectively monitorized organ function and performed skin biopsy during the ICU stay, and evaluated the survived patients at follow-up with focused neurological examination. Our results provide the first-ever evidence that critical illness causes also small nerve fiber degeneration since the early phases of the disease.

## Materials and Methods

Critically ill neurologic patients admitted to the Neurocritical Care Unit of the University of Brescia at the Spedali Civili of Brescia, Italy, from September 1, 2011 to May 1, 2012 were consecutively recruited. Inclusion criteria were age ≥18 years, ICU stay ≥ 3 days, and need of either invasive or non-invasive ventilatory support. Criteria for exclusion were ascertained after discussion with patient’s family physician and family members, and after careful investigation of the clinical charts of previous hospital admissions. Exclusion criteria included the following: any previously known neuromuscular disease or condition at risk for peripheral neuropathy, such as diabetes, systemic diseases with renal, hepatic or thyroid dysfunction, alcohol abuse, HIV or hepatitis C infection, hematological diseases or monoclonal gammopathy of uncertain significance, vitamin B_12_ deficiency, connective tissue diseases (e.g. Sjögren syndrome), malignancies, previous or current treatment with neurotoxic drugs (e.g. antineoplastic or anti-arrhythmic drugs). We also excluded patients with chronic pain, those in terminal conditions and those in whom skin biopsy could not be taken or electrophysiological investigations of peripheral nerves and muscles were not feasible (e.g. coagulation disorders, plasters, limb amputation, limb edema, etc.) [20].

### Ethics statement

The study was approved by the Ethics Committee of the University of Brescia at Spedali Civili (CRitical Illness Neuropathy of Epidermal fibers - CRINE study doc n.371/gt). Because all patients had brain injury and altered consciousness at ICU admission, the Ethics Committee permitted to use waived consent. In fact, the definition of the legal representative, or surrogate, of adult temporarily incapacitated ICU patients is absent in Italy [21]. Detailed written information was provided to the family members about the study protocol, the need for and safety of minimally invasive skin biopsy and the need of follow-up visits. At follow-up, written informed consent was obtained from the patients if they had regained their mental competency. All clinical investigations were conducted according to the principles expressed in the Declaration of Helsinki.

### General, neurological, and electrophysiological assessment

During the ICU stay, patients were followed daily for the development of sepsis or multiple organ failure (MOF). Sepsis was defined according to current recommendations as the presence of both infection and systemic inflammatory response [22]. MOF was diagnosed as the evidence of two or more organ failures according to the sequential organ failure assessment score [23].

As critically ill patients often develop acute sensory-motor axonal polyneuropathy and/or myopathy, we performed nerve conduction studies (NCS) and needle electromyography (EMG). We adopted currently recommended criteria to diagnose critical illness polyneuropathy (CIP) and critical illness myopathy (CIM) [3] using conventional techniques. Manual muscle strength testing was not possible during the ICU stay due to the altered consciousness of patients. Hence, we defined CIP and CIM as “probable” based on previously proposed criteria [3].

At follow-up, all patients underwent neurological examination including evaluation of consciousness, sensory functions, and muscle strength when permitted by the patient clinical conditions. Signs suggestive of autonomic dysfunction (e.g. abnormal sweating, lacrimation and/or salivation; gastrointestinal, bladder, and sexual disorders; orthostatic hypotension) were recorded. The finger wrinkling test was recorded after immersion of the right hand in a bucket filled with water at 40 °C and used to investigate sympathetic peripheral dysautonomia [24]. The degree of skin wrinkling at the fingertip was assessed at baseline and after 5, 15, and 30 minutes, and graded using a semi-quantitative 5-point clinical scale. We scored 0 in case of absent wrinkling, 1 when the skin was not completely smooth, 2 when two or less lines of wrinkling were seen, 3 when three or more lines of wrinkling were seen, and 4 when wrinkling completely distorted the pulp of the fingertip. The scores from 0 to 2 were considered abnormal (e.g. peripheral dysautonomia), whereas scores ≥3 were considered normal [25]. Severity of spontaneous and evoked pain (tactile allodynia and hyperalgesia) was graded using the 11-point Likert numerical rating score (NRS).

The modified Rankin Scale (mRS) was used to categorize the outcome as follow: mRS; 1= no symptoms; 2 or 3= moderate disability, able to walk but unable to perform all activities independently; 4= moderate-to-severe disability, unable to walk independently; 5= severe disability, bed-bound; 6= dead.

### Skin biopsy

Skin biopsies were performed with a minimally invasive technique [8] as soon as the general condition stabilized (e.g. when there was no evidence of intracranial hypertension; coagulation disorders; hemodynamic instability requiring inotropic support; respiratory failure requiring mandatory mechanical ventilation; kidney insufficiency requiring dialysis; fever or sepsis). Biopsies were taken using a 3-mm disposable punch, after local anesthesia with 2% lidocaine under sterile technique, at the distal site of the leg (10 cm above the lateral malleolus within the territory of the sural nerve) and at the proximal site on the thigh (20 cm below the iliac spine). Specimens were immediately fixed for 24 hours at 4°C, cryoprotected, and shipped to the Skin Biopsy Laboratory at the “Carlo Besta” Neurological Institute of Milan, Italy, to be processed. Three sections randomly chosen from each sample were immunoassayed with polyclonal anti-protein gene product (PGP) 9.5 antibodies (Biogenesis, Poole, Dorset, UK; 1:1000) using the free-floating protocol for bright-field immunohistochemistry previously described [26]. The linear density of IENF was calculated according to current guidelines [17]. Densities from patients’ distal leg biopsy samples were compared with age- and gender-adjusted normative values [17], whereas those from the proximal thigh were compared with archive normative values [9]. Sweat gland innervation was assessed using a semi-quantitative approach comparing the density of PGP9.5-positive fibers around ducts with archive biopsy sections from healthy subjects. Sweat gland were considered completely or partially denervated when no or only sparse PGP 9.5-positive fibers, respectively, were recognizable.

### Data presentation

Data were reported as means and standard deviation (SD) when normally distributed, and median with 25 to 75% quartiles (interquartile range, IQR) when not normally distributed.

## Results

During the study period, 246 patients were excluded because the ICU stay was less than 3 days (158 patients), or patients had diabetes (41 patients), malignancy (19 patients), alcohol abuse (7 patients), renal insufficiency (4 patients), liver cirrhosis (4 patients), pre-existing neuromuscular disorders (3 patients), hepatitis C infection (2 patients), patients were in terminal condition (7 patients) or skin biopsy was not feasible (1 patients). Fourteen consecutive patients (5.7%) were eventually recruited.

Demographic characteristics, admission diagnoses, NCS, EMG, and skin biopsy findings are detailed in Table 1. Median duration of mechanical ventilation was 12 days (IQR 8-15). Median ICU stay was 17 days (IQR 11-18). Thirteen patients developed sepsis during the ICU stay, predominantly related to respiratory, urinary, and bloodstream infections. Only one did not have proven infection despite persistent fever.

**Table 1:** Patients’ characteristics.

Patient		Admission diagnosis	Admission GCS	ICU complications	Nerve conduction study	IENF per mm (lower limit of normality)		Sweat gland innervation
No.	Gender, age		Eye-Motor-Verbal			proximal thigh	distal leg	
1	M, 55 y	Subarachnoid hemorrhage	1-6-1	VAP, UTI, CRBSI, sepsis	Axonal sensory-motor neuropathy	4.3 (12.8)	2.6 (3.5)	Reduced
2	M, 48 y	Head trauma	2-5-1	VAP, sepsis	Myopathy	5.6 (12.8)	2.7 (4.4)	Reduced
3	M, 74 y	Hemorrhagic shock, hypotensive encephalopathy	2-1-1	VAP, septic shock	Axonal sensory-motor neuropathy	0 (12.8)	1.2 (2.1)	Reduced
4	M, 56 y	Subarachnoid hemorrhage	1-1-1	VAP, sepsis, SE	Axonal sensory-motor neuropathy	6.3 (12.8)	2.6 (3.5)	Reduced
5	F, 47 y	Intracerebral hemorrhage	3-6-1	VAP, sepsis	Axonal sensory-motor neuropathy	6.3 (12.8)	1.7 (5.7)	Reduced
6	M, 56 y	Subarachnoid hemorrhage	1-5-1	VAP, UTI, CRBSI, sepsis, MOF, DCI-CI	Myopathy	7.2 (12.8)	1.9 (3.5)	Normal
7	F, 66 y	Ischemic stroke	4-5-1	VAP, CRBSI, sepsis, MOF	Myopathy	1.6 (12.8)	0.2 (3.2)	Reduced
8	F, 77 y	Subarachnoid hemorrhage	3-6-3	EVD-meningitis, sepsis, MOF	Myopathy	7.0 (12.8)	1.4 (2.2)	Reduced
9	M, 62 y	Subarachnoid hemorrhage	3-6-1	Central hyperthermia, DCI-CI	Normal findings	0 (12.8)	0 (2.8)	Reduced
10	F, 71 y	Neurosurgery (pituitary adenoma)	3-6-2	Cerebral ischemia	Myopathy	2.2 (12.8)	0.6 (2.2)	not available
11	M, 45 y	Cranio-facial trauma	1-5-2	VAP, septic shock	Normal findings	2.5 (12.8)	1.7 (4.4)	Reduced
12	F, 77 y	Subdural abscess	1-5-1	Sepsis	Myopathy	3.0 (12.8)	1.7 (2.2)	Reduced
13	F, 41 y	Subarachnoid hemorrhage	1-3-1	Meningitis, VAP, sepsis	Myopathy	4.6 (12.8)	3.7 (5.7)	Reduced
14	F, 58 y	Neurosurgery (meningioma)	3-6-1	UTI, sepsis	Myopathy	1.1 (12.8)	1.3 (4.3)	Reduced

GCS, Glasgow Coma Scale. ICU, intensive care unit. IENFD, intraepidermal nerve fibers; mRS, modified Rankin Scale: 0= no symptoms; 1= significant disability despite symptoms; 2, 3= mild or moderate disability; 4= moderate-to-severe disability; 5= severe disability; 6=dead. VAP: ventilator-associated pneumonia; UTI: urinary tract infection; CRBSI: catheter-related bloodstream infection. SE: status epilepticus. EVD-meningitis: meningitis associated with external ventricular drainage. DCI-CI: delayed cerebral ischemia with cerebral infarction.

NCS study and EMG were performed on median ICU day 8 (IQR 7-14 days) and revealed CIM in 8 patients, CIP in 4 patients, and normal findings in 2 patients.

**Figure 1:**
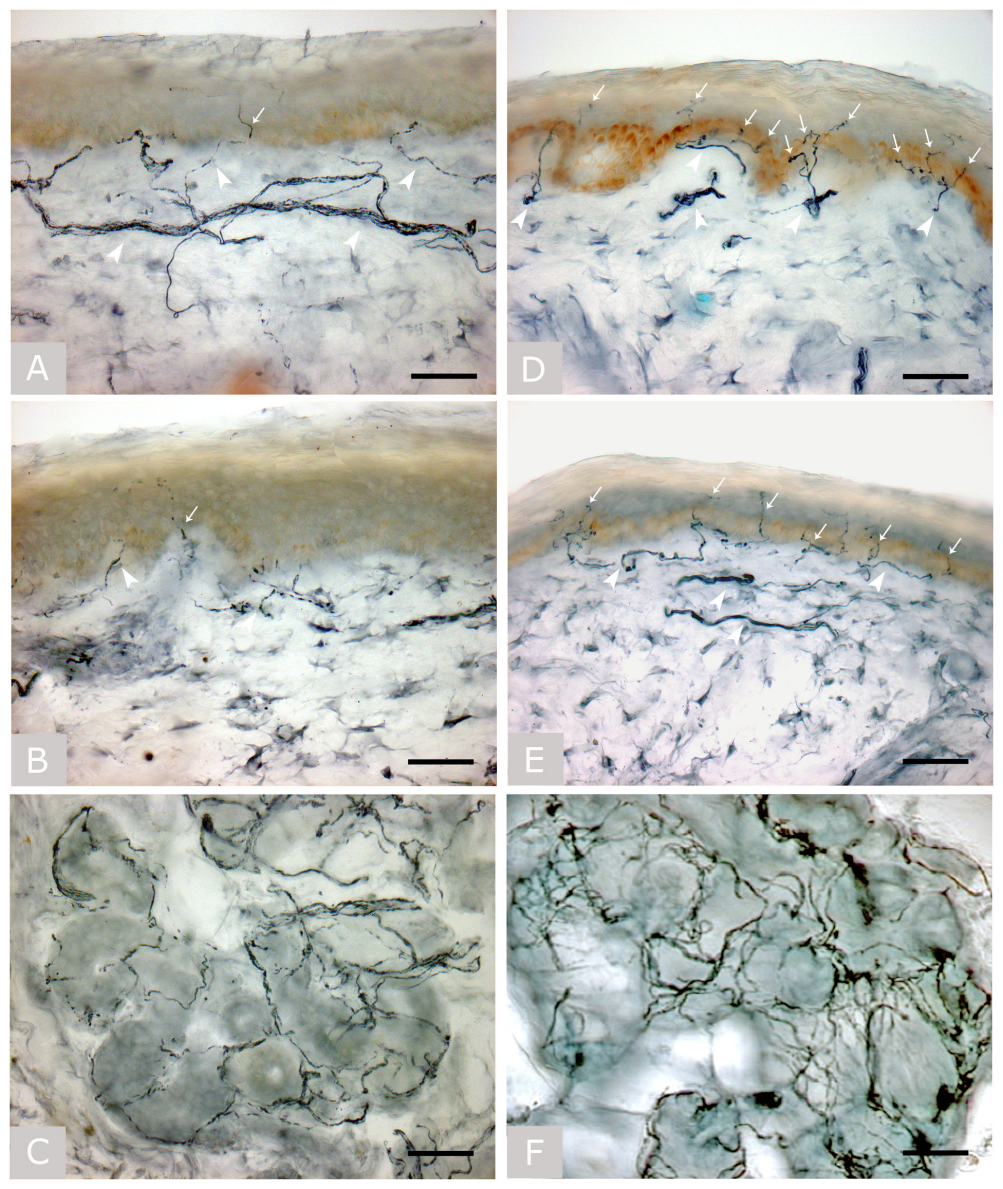
Skin biopsy. Bright field immunohistochemical studies (polyclonal anti-protein gene product 9.5 antibody; Ultraclone, Wellow, Isle of Wight, UK) in sections of skin biopsy taken at the proximal thigh (A) and distal site of the leg (B) with a sweat gland (C) in patient no. 7, and at the proximal thigh (D) and distal site of the leg (E) with a sweat gland (F) in a healthy subject. Arrows indicate intra-epidermal nerve fibers and arrowheads indicate dermal nerve bundles. The patient, with normal nerve conduction study findings and myopathy at needle electromyography, showed a severe depletion of intra-epidermal nerve fibers and a severe reduction in the density of dermal nerve bundles, whose staining was fragmented and weaker due to axonal degeneration. Sweat gland innervation was also markedly reduced. Original magnification 40x. Bar is 50 µm in all images.

Skin biopsies were obtained on median ICU day 22 (IQR 18-24 days). All patients showed a severe and non-length dependent degeneration of IENF with densities below normal values at both distal leg and proximal thigh (table **1**). Epidermal and dermal nerves showed diffuse degenerative changes with fragmented and weak staining. Sweat glands were severely denervated in all except one patient with CIM (Figure **1**). There were no complications associated with skin biopsies.

Two patients died after ICU discharge. At follow-up, 2 patients regained full functional independence, 1 patient had mild disability, 4 patients had moderate-to-severe disability, and 5 patients had severe disability (table **2**). Seven patients (no. 2, 5, 7, 8, 11, 12, 14) were available for follow-up examination and three of them complained of sensory loss and burning pain (no. 2, 8 and 12). Pain was diffuse, and in one patient (no. 2) it was so severe to interfere with effective physical rehabilitation. Intriguingly, all these 3 patients had acute myopathy with no evidence of large fiber sensory neuropathy at NCS. Their sensory symptoms likely reflected a small fiber neuropathy as diagnosed by skin biopsy.

**Table 2:** Patients’ neurological findings at follow-up visit.

**Patient**	**Sensory signs**	**Motor signs**	**Dysautonomic signs**	**Finger wrinkling test**	**Follow-up** (days after ICU discharge)	**GCS**	**Outcome mRS**
**No.**						Eye-Motor-Verbal	
1	NA	NA	NA	NA	6 months	/	6
2	Sensory loss, severe paroxysmal pain (NRS 9/10) and continuous burning pain (NRS 3/10) in lower limbs; dynamic mechanic allodynia	Spastic paraparesis	None	3	13 months	15	4
3	NA	NA	NA	NA	9 months	/	6
4	Grimacing and limb movements after pain stimulation of lower limbs; no reaction after upper limb stimulation	Spontaneous limb movements, pyramidal signs.	None	3	9 months	4-5-1 (global aphasia)	5
5	None	Left hemiparesis	None	4	9 months	15	3
6	Not assessable	Spastic tetraplegia	None	3	8 months	4-3-1	5
7	None	None	None	3	10 months	15	1
8	Sensory loss, burning pain (NRS 5/10)	Spastic paraplegia, upper limb weakness	None	NA	10 months	4-6-2	5
9	Not assessable	Spastic tetraplegia	Orthostatic hypotension, tachycardia, abnormal sweating, clammy skin	1	10 months	4-2-1 (VS)	5
10	Not assessable	Flaccid tetraplegia	Orthostatic hypotension, tachycardia, abnormal sweating, clammy skin	NA	10 months	4-1-1 (MCS)	5
11	None	None	None	NA	10 months	15	1
12	Light touch, pinprick, and vibratory sensory loss with stocking-glove distribution; burning pain at distal lower limbs (NRS 4/10)	Reduced foot dorsiflexion	None	3	9 months	15	3
13	Grimacing after pain stimulation of upper and lower limbs	Upper limb extension and lower limb flexion after painful stimulation	Tachycardia, abnormal sweating	NA	8 months	4-2-1 (MCS)	5
14	None	Right hemiparesis	None	4	8 months	15	4

Finger wrinkling test: scores from 0 to 2 indicate abnormal skin wrinkling; score of 3 or 4 indicate normal skin wrinkling. GCS, Glasgow Coma Scale. mRS, modified Rankin Scale: 0= no symptoms; 1= no significant disability despite symptoms; 2, 3= mild or moderate disability; 4= moderate-to-severe disability; 5= severe disability; 6=dead. VS, vegetative state. MCS, minimally conscious state.

Clinical dysautonomia was observed in 3 patients, of whom 1 (no. 9) also had abnormal finger wrinkling test (table **2**). The only patient with normal density of sweat gland nerve fibers at skin biopsy (no. 6) did not show any sign of systemic, cardiovascular, and peripheral dysautonomia at clinical evaluation and finger wrinkling test.

## Discussion

We report the first-ever observation that critical illness can be associated with a non-length dependent degeneration of somatic and autonomic small nerve fibers. This complication developed in critically ill patients with severe infection, sepsis or MOF, and prolonged mechanical ventilation and ICU stay. In association with the degeneration of IENF, patients showed also electrophysiological evidence of acute axonopathy of large nerve fibers and acute myopathy, confirming that neuromuscular alterations of critical illness complicate the clinical course of the most severely ill patients and are not isolated events, but rather are an integral part of the process leading to multiple organ dysfunctions and failures [3].

IENF degeneration has been described in several chronic conditions, such as diabetic neuropathy [27,28], but also in acute inflammatory neuropathies, such as Guillain-Barré syndrome [11-14]. Patients with SFN typically present with neuropathic pain and reduced thermal and pain sensation but with normal strength, proprioceptive sensation, and deep tendon reflexes reflecting normal large nerve fiber functions [9,10,29,30]. At skin biopsy, reduced IENF density is typically demonstrated [30]. In our series, we found a non-length dependent degeneration of somatic IENF in all the patients, suggesting that critical illness likely affects dorsal root ganglion (DRG) neurons. Patients available for neurological examination at follow-up complained of diffuse pain and thermal and nociceptive sensory loss, a pattern typical of sensory neuronopathies [18]. DRG neurons are surrounded by fenestrated capillaries instead of a tight barrier and are therefore exposed to circulating neurotoxic factors from the bloodstream. Circulating mediators such as endotoxin, other proinflammatory agents (tumor necrosis factor-a, serotonin, and histamine) or neurotoxic factors are probably released during sepsis or systemic inflammatory response syndrome, and have been proposed in the pathogenesis of CIP, but their role remains controversial because CIP can occur in the absence of an identifiable circulating mediator [31]. Future studies should investigate these candidate neurotoxic factors for their effects on the tiny nerve fibers of the skin.

Our analyses of skin biopsies were aimed at describing morphometric and morphologic features of epidermal and dermal nerves, and did not include immunohistochemical assays of cytokines or adhesion molecule expression, which might provide clue to the underlying pathogenetic mechanisms of IENF degeneration. Increased production of pro-inflammatory cytokines has a key role in promoting the organ dysfunction during the early phase of critical illness [32], and may also increase the microvascular nerve permeability leading to formation of endoneurial and axon disruption [3,33]. Since all our patients developed infections and sepsis during the ICU stay, we hypothesize that the degeneration of IENF was caused by the same mechanisms leading to axonal degeneration of large nerve fibers and multiple organ dysfunction. This hypothesis is further supported by the strict inclusion criteria adopted, which allowed us to exclude patients at risk for neuropathy before ICU admission. In addition, we performed all skin biopsies during the ICU stay over a relatively short period of time in order to detect potential risk factors with high accuracy. Future studies should evaluate if skin accumulation of inflammatory cytokines or increased expression of adhesion molecules might play a role in IENF degeneration during critical illness.

Neuropathic pain, defined as pain caused by a disease or a lesion of the somatosensory nervous system [34], was observed in 3 patients who showed thermal and nociceptive sensory loss and burning pain associated with IENF loss. This picture was likely caused by the exclusive degeneration of small nerve fibers, because NCS were normal, therefore excluding CIP or other large fiber neuropathies. Besides degeneration of the somatic IENF, all except one patient showed a profound denervation of skin sweat glands indicating autonomic peripheral involvement. Autonomic dysfunctions were evident in 3 patients, one of whom also had abnormal finger wrinkling test. However, the impact of these abnormalities on patients’ outcome could not be assessed due to severe neurologic conditions. This result confirms previous report of autonomic dysfunction with abnormal cardiac R-R variation and sympathetic skin response in ICU patients with critical illness polyneuropathy or septic encephalopathy [35].

One major limitation of our study is the small sample of patients with no control group and the fact that accurate neurological evaluation of the sensory system at follow-up was possible only in a subset of patients. Future studies should involve larger samples of non-neurologic critical care patients. In future follow-up studies, serial skin biopsies could also be offered to patients to evaluate the natural progression of the disease and the effect of potential treatments [36]. Finally, inclusion of patients without sepsis and MOF would help to confirm if IENF degeneration is part of the process leading to MOF.

Our study could contribute in addressing some issues regarding the long-term outcome of ICU survivors. In particular, chronic pain is common and probably involves multiple pathogenetic mechanisms [6,37,38]. Our findings suggest that the degeneration of somatic and autonomic small nerve fibers is a frequent complication of critical illness and explained the persisting sensory deficits and neuropathic pain observed in some survivors. In such cases, skin biopsy is a safe, almost painless, and cheap technique that can be offered to patients for evaluating small nerve fibers.
